# Kansas City

**Published:** 1896-04

**Authors:** 


### KANSAS CITY VETERINARY COLLEGE.

THE fifth annual commencement exercises were held in the Masonic Building, 912 Walnut Street, on March 19th. The address to the students was delivered by Professor Isadore Wolf. Professor Wattles delivered the diplomas and conferred the degree of D.V.S. on the following: Frank H. Ames, Waverly, Mo.; Albert A. Immel, Springfield, Mo.; William A. Cock, Clinton, Mo.; Charles F. Hickman, Galena, Kan.; Edward L. McCrea, Lamar, Mo.; Elwood J. Netherton, Gallatin, Mo.; Samuel Sheldon, Turney, Mo.; William N. Hobbs, Kensington, Kan.; James C. Keely, Waterford, Ireland.

